# Concentration-Dependent Modulation of Fibrillated Egg White Protein by γ-Cyclodextrin: Structural, Emulsifying, and Rheological Properties of Oil-in-Water Emulsions

**DOI:** 10.3390/foods15183168

**Published:** 2026-09-08

**Authors:** Xiaomeng Li, Mohamed Salama, Tamer M. El-Messery

**Affiliations:** 1 School of Life Sciences and Health Engineering, Luoyang Institute of Science and Technology, Luoyang 471023, China; 2 Department of Food Science and Engineering, School of Agriculture and Biology, Shanghai Jiao Tong University, Shanghai 200240, China; mohamed.salama@webmail.hzau.edu.cn; 3 Dairy Department, Food Industries and Nutrition Research Institute, National Research Centre, Dokki, Cairo 12622, Egypt; 4 Department of Food Science and Human Nutrition, College of Agriculture and Food, Qassim University, Buraydah 51452, Saudi Arabia

**Keywords:** egg white protein fibrils, γ-cyclodextrin, O/W emulsions, emulsifying properties, rheology, protein function

## Abstract

Fibrillated egg white protein (FEWP) has promising emulsifying functionality, whereas excessive association may restrict the organization of freshly prepared emulsions. This study examined the concentration-dependent effects of γ-cyclodextrin (γ-CD; 0.5%, 1%, and 2%, *w*/*v*) on 3% (*w*/*v*) FEWP dispersions and the resulting oil-in-water emulsions. Relative to native egg white protein (NEWP), redispersed FEWP showed a smaller apparent hydrodynamic diameter (36.56 vs. 371.97 nm). γ-CD caused only minor changes in particle size and a non-monotonic intrinsic-fluorescence response. Among the γ-CD levels tested, 1% produced the highest intrinsic fluorescence intensity, emulsifying activity index (2.56 m^2^/g), and emulsion stability index (207.87 min). In the observed microscopic fields, this formulation contained fewer conspicuous large droplets and a visually more uniform spatial distribution than the other FEWP-γ-CD formulations. It also moderated progressive microscopic immobilization and maintained elastic-dominated, shear-thinning, and partially recoverable behavior. Increasing γ-CD to 2% reduced emulsifying performance and bulk structural connectivity. Thus, 1% γ-CD provided the most favorable combination of short-term emulsifying and rheological properties among the concentrations examined.

## 1. Introduction

Egg white protein (EWP) is a nutritionally valuable food protein with versatile techno-functional properties, including gelation, foaming, and emulsification, making it an important ingredient in food colloids and multiphase systems [1]. However, the compact globular structures of native egg white proteins (NEWPs) restrict the accessibility of internal hydrophobic residues and may limit protein diffusion, unfolding, and rearrangement at oil–water interfaces [2]. Acid/heat-induced fibrillation offers an effective route for overcoming these structural constraints. During prolonged heating under acidic conditions, proteins undergo partial hydrolysis, conformational unfolding, and ordered self-assembly into fibrillar aggregates enriched in cross-β-sheet structures [3]. Compared with their native counterparts, these elongated structures possess larger specific surface areas, greater exposure of hydrophobic regions, and more accessible sites for intermolecular and interfacial interaction [4,5]. Protein fibrils may therefore stabilize emulsions through both adsorption at the oil–water interface and the formation of fibrillar networks in the continuous phase, thereby restricting droplet migration, flocculation, and coalescence [6].

Nevertheless, fibrillation does not invariably improve emulsifying performance [7]. Excessive exposure of hydrophobic regions can promote nonspecific fibril association and generate heterogeneous aggregates before adsorption at newly created interfaces [8]. Strong fibril–fibril interactions may also induce bridging between oil droplets, whereas highly associated fibrils may exhibit reduced mobility during homogenization [9,10]. Consequently, the functionality of fibrillated proteins depends on a balance among hydrophobic accessibility, aqueous dispersibility, interfacial affinity, and network-forming capacity rather than on fibril formation or surface hydrophobicity alone. Thus, regulating excessive fibrillar association while retaining sufficient affinity for the oil phase is critical for the rational design of physically stable fibril-based emulsions.

Cyclodextrins are cyclic oligosaccharides characterized by a hydrophilic outer surface and a relatively hydrophobic internal cavity, enabling them to associate with suitably sized nonpolar moieties through inclusion and other non-covalent interactions [11,12]. γ-Cyclodextrin (γ-CD), composed of eight glucose units, has a larger cavity than α- and β-cyclodextrins and may accommodate relatively bulky hydrophobic groups or molecular segments. In protein systems, cyclodextrins can interact with solvent-accessible aromatic residues, including tryptophan, tyrosine, and phenylalanine, and may thereby alter their local solvent environment and association behavior [13]. γ-CD may therefore alter the colloidal characteristics of fibrillated egg white protein (FEWP) and its subsequent emulsion-forming behavior, potentially in a concentration-dependent manner. However, although cyclodextrin–protein interactions have been investigated in native globular proteins, model peptides, and small-molecule inclusion systems [11,13,14], the regulation of food-protein fibril systems by γ-CD remains poorly understood. In particular, the relationships among γ-CD concentration, the colloidal state and aromatic-residue microenvironment of FEWP, and the emulsifying and rheological properties of freshly prepared FEWP emulsions, have not been systematically evaluated. Importantly, any observed changes can indicate concentration-dependent associations but do not, by themselves, establish specific host–guest binding or interfacial rearrangement.

Accordingly, this study investigated the concentration-dependent effects of γ-CD on fibrillated EWP and on the short-term emulsifying and rheological properties of the resulting oil-in-water emulsions. It was hypothesized that an intermediate γ-CD concentration would moderate excessive FEWP association while retaining sufficient interfacial functionality. Colloidal and spectroscopic characterization of the aqueous systems was integrated with measurements of the emulsifying activity index (EAI) and emulsion stability index (ESI), qualitative microscopy, microrheology, bulk rheology, creep-recovery, and three-interval thixotropy. By relating changes in the FEWP dispersion state to emulsion performance, this work provides a physicochemical basis for the rational application of γ-CD in FEWP-based emulsion systems.

## 2. Materials and Methods

### 2.1. Materials

Fresh eggs from Hy-Line Brown laying hens were purchased from a local supermarket and used for EWP preparation. γ-CD was supplied by Shandong Zibo Qianhui Co., Ltd. (Zibo, China). The corn germ oil (Arawana brand, Yihai Kerry Arawana Holdings Co., Ltd., Shanghai, China) was purchased from a local supermarket. Bovine serum albumin, used as the protein standard, was purchased from BioFroxx-Sai Guo Biotechnology Co., Ltd. (Guangzhou, China). HCl and sodium dodecyl sulfate were supplied by Sinopharm Chemical Reagent Co., Ltd. (Shanghai, China). Unless otherwise stated, all chemicals were of analytical grade, and ultrapure water generated using a Milli-Q water purification system (Merck Millipore, Darmstadt, Germany; nominal resistivity: 18.2 MΩ·cm; nominal conductivity: 0.055 μS/cm at 25 °C) was used to prepare all solutions.

### 2.2. Preparation of Fibrillated Egg White Protein (FEWP)

FEWP was prepared following a procedure previously established by our group, with appropriate modifications [3]. Fresh egg white was separated from the yolk and combined with an equal volume of ultrapure water. The pH of the diluted egg white was adjusted to 5.0 with 6 mol/L HCl using an FE20 pH meter (Mettler-Toledo, Shanghai, China), followed by magnetic stirring for 30 min using a DF-101S magnetic stirrer (Shanghai Weikai Instrument Equipment Co., Ltd., Shanghai, China). Insoluble components were removed by centrifugation at 5500× *g* for 15 min at 4 °C in a refrigerated centrifuge (CR22N, Hitachi, Tokyo, Japan). The protein content of the recovered supernatant was determined by the Biuret method using bovine serum albumin as the standard. The dispersion was subsequently diluted with ultrapure water to a protein concentration of 2% (*w*/*v*), and its pH was adjusted to 2.0 with 6 mol/L HCl. Fibrillation was induced by heating the dispersion at 85 °C for 8 h in a thermostatically controlled water bath (HH-2, Changzhou Guohua Electric Appliance Co., Ltd., Changzhou, China). Immediately after heating, the samples were cooled in an ice water bath to arrest further structural evolution. In our previous study, transmission electron microscopy confirmed fibrillar morphology in the acid heat-treated dispersion [15]. The cooled fibrillar dispersion was converted into powder using a laboratory-scale spray dryer (SP-1500, Shanghai Shunyi Experimental Equipment Co., Ltd., Shanghai, China). The inlet air temperature was maintained at 180 °C, and the feed rate was 300 mL/h. The collected FEWP powder was sealed in airtight containers and stored at −20 °C until further use.

### 2.3. Preparation of FEWP-γ-CD Complexes

FEWP powder was reconstituted in ultrapure water to obtain a 6% (*w*/*v*) protein stock dispersion and stirred until complete hydration. Separately, γ-CD solutions were prepared at 1%, 2%, and 4% (*w*/*v*). Equal volumes of the FEWP stock and the corresponding γ-CD solutions were then combined, yielding formulations containing 3% (*w*/*v*) FEWP with 0.5%, 1%, or 2% (*w*/*v*) γ-CD. The mixtures were incubated under continuous magnetic stirring at 25 °C for 2 h to promote equilibration between the two components. The FEWP control was prepared by diluting the 6% (*w*/*v*) stock dispersion twofold with ultrapure water, giving a final protein concentration of 3% (*w*/*v*). NEWP was adjusted to the same final concentration and processed under identical conditions. A 2% (*w*/*v*) γ-CD solution without protein was included as the γ-CD control. All formulations were freshly prepared and subjected to the same handling conditions before analysis. The ultrapure water used for preparation and dilution had a measured pH of 7.0.

### 2.4. Particle Size and Zeta Potential

Particle-size characteristics and electrophoretic mobility were determined using a Zetasizer Nano ZS analyzer (Malvern Instruments Ltd., Malvern, Worcestershire, UK), following Li et al. [14] with minor modifications. To minimize multiple scattering and concentration-dependent effects, protein-containing dispersions were diluted with the same ultrapure water (pH 7.0) to a final protein concentration of 1 mg/mL; no pH adjustment was made after dilution, and the final dispersion pH was not recorded. The γ-CD control underwent the same volumetric dilution. For particle-size analysis, samples were loaded into disposable polystyrene cuvettes, equilibrated at 25 °C, and analyzed by dynamic light scattering (DLS) to obtain the Z-average diameter, polydispersity index (PDI), and intensity-weighted size distribution. Electrophoretic mobility was measured at 25 °C in folded capillary cells (DTS1070). The instrument software (Zetasizer Software Version 8.02) converted mobility to an estimated zeta potential using the Smoluchowski approximation to Henry’s equation [16,17]. Three independently prepared samples were analyzed for each formulation.

### 2.5. Ultraviolet–Visible (UV–Vis) Spectra

Ultraviolet absorption spectra were recorded using a NanoDrop 2000C UV-Vis spectrophotometer (Thermo Fisher Scientific, Wilmington, DE, USA). Before analysis, the protein-containing samples were diluted with ultrapure water to a final protein concentration of 1 mg/mL, while the γ-CD control was subjected to the same volumetric dilution. The diluted samples were transferred into quartz cuvettes with a 10 mm optical path length, and spectra were collected from 240 nm to 340 nm at room temperature using ultrapure water as the reference blank. Changes in absorbance and spectral features in the aromatic amino acid region were examined to evaluate alterations in the local environments of phenylalanine, tyrosine, and tryptophan residues. After appropriate smoothing, the second-derivative spectra were calculated from the original absorption spectra using OriginPro 2026 software (OriginLab Corporation, Northampton, MA, USA) [18].

### 2.6. Intrinsic Fluorescence Spectrum

Intrinsic fluorescence spectra were recorded using an F-4600 fluorescence spectrophotometer (Hitachi, Tokyo, Japan) to evaluate changes in the microenvironment of aromatic amino acid residues. Protein-containing dispersions were diluted with ultrapure water to a final protein concentration of 0.2 mg/mL, while the γ-CD control was subjected to the same volumetric dilution. Samples were placed in quartz fluorescence cuvettes with a 10 mm optical path length and excited at 290 nm, and emission spectra were collected from 300 nm to 450 nm at room temperature. Both the excitation and emission slit widths were set to 5 nm. After subtraction of the corresponding blank spectrum, the maximum fluorescence intensity was obtained to characterize changes in the local environment of tryptophan residues [19].

### 2.7. Preparation of Oil-in-Water Emulsions

The dispersions described above were used as the aqueous phase for emulsion preparation. Corn germ oil was added gradually to each aqueous formulation to give an aqueous-to-oil volume ratio of 70:30 [15]. The mixtures were homogenized at 10,000 rpm for 2 min using an XHF-DY high-speed homogenizer (Ningbo Scientz Biotechnology Co., Ltd., Ningbo, China) to produce oil-in-water emulsions. NEWP, FEWP, FEWP-γ-CD complexes, and γ-CD alone were processed under the same conditions. The freshly prepared emulsions were immediately subjected to physicochemical, microstructural, and rheological characterization.

### 2.8. Emulsifying Properties

The emulsifying activity index (EAI) and emulsion stability index (ESI) were determined from the turbidity of diluted emulsions [15]. Immediately after homogenization, 100 μL of emulsion was withdrawn and dispersed in 3.9 mL of 0.1% (*w*/*v*) sodium dodecyl sulfate solution. The mixture was gently vortexed (XW-80A, Haimen Kylin-Bell Lab Instruments Co., Ltd., Haimen, China), and its absorbance at 500 nm was recorded using the NanoDrop 2000C UV-Vis spectrophotometer (Thermo Fisher Scientific, Wilmington, DE, USA). A second aliquot was analyzed in the same manner after the emulsion had remained undisturbed at room temperature for 30 min. EAI and ESI were calculated as follows:

(1) $$\mathrm{EAI}\left(m^{2}/g\right)=2\times 2.303\times A_{0}\times N/(C\times \phi \times (1-\phi )\times 10^{4})$$

(2) $$\mathrm{ESI}\left(\min\right)=A_{0}\times \Delta t/\left(A_{0}-A_{T}\right)$$

where $A_{0}$ is the absorbance measured immediately after emulsification, $A_{T}$ is the absorbance measured after the designated storage period, N is the dilution factor, C is the protein concentration in the aqueous phase (g/mL), ϕ is the oil volume fraction, and Δt is the time interval between the two measurements. In this study, N, ϕ, and Δt were 40, 0.30, and 30 min, respectively. Three independently prepared emulsions were analyzed for each formulation.

### 2.9. Microscopic Observation

The microstructure of the freshly prepared emulsions was examined using an EX20 biological microscope (Sunny Optical Technology (Group) Co., Ltd., Ningbo, China). A small aliquot of each emulsion was placed on a clean glass slide and carefully covered with a coverslip to minimize compression-induced structural changes. Images were acquired at 40× magnification under identical illumination and imaging conditions. Multiple fields of view were examined for each formulation to evaluate differences in droplet distribution, aggregation, and spatial homogeneity, and representative micrographs were presented.

### 2.10. Microrheological Measurements

The time-dependent microrheological properties of the emulsions were monitored using a Rheolaser Master analyzer (Formulaction, L’Union, France) based on multi-speckle diffusing wave spectroscopy. Fresh emulsion (4 mL) was gently introduced into a cylindrical glass cell, with care taken to avoid bubble formation and additional shear disturbance. The cell was maintained at 25 °C in the measuring chamber, and the sample was monitored under quiescent conditions for 1 h. Changes in the backscattered speckle pattern caused by the microscopic motion of the dispersed entities were processed to generate mean square displacement (MSD) curves. The solid–liquid balance (SLB) and fluidity index (FI) were subsequently obtained from the instrument software to describe the evolution of emulsion mobility and viscoelastic behavior [20].

### 2.11. Rheological Measurements

Rheological measurements were carried out at 25 °C using a DHR-2 rotational rheometer (TA Instruments, New Castle, DE, USA) fitted with a 40 mm aluminum parallel plate. The linear viscoelastic range of each emulsion was established from a preliminary amplitude sweep. A strain of 1%, confirmed to lie within this range, was then selected for the frequency sweep. Changes in the storage modulus (G’) and loss modulus (G″) were monitored as the angular frequency increased from 0.1 to 100 rad s^−1^. In a separate flow test, the apparent viscosity was recorded over a shear-rate range of 0.01–100 s^−1^ to describe the steady-shear response of the emulsions [21].

### 2.12. Creep-Recovery and Thixotropic Behavior

Structural breakdown and rebuilding under changing shear conditions were examined through a three-step shear sequence. The emulsions were initially maintained at 0.1 s^−1^ for 120 s, exposed to 100 s^−1^ for the next 120 s, and finally returned to 0.1 s^−1^ for another 120 s. Apparent viscosity was continuously monitored to follow the loss and restoration of the internal structure. Creep-recovery behavior was determined according to a previously reported procedure [22]. The samples were subjected to a constant stress for 180 s and subsequently allowed to recover for 360 s after the stress was released. The strain profiles obtained during both stages were used to compare the resistance to deformation and the extent of structural recovery among the emulsions [21].

### 2.13. Statistical Analysis

Each formulation was independently prepared three times, and the results are reported as the mean ± standard deviation. Statistical differences among formulations were evaluated by one-way analysis of variance (ANOVA) followed by Duncan’s multiple range test using IBM SPSS Statistics 27.0 (IBM Corp., Armonk, NY, USA). Differences were considered statistically significant at *p* < 0.05. Figures were prepared using OriginPro 2026 (OriginLab Corporation, Northampton, MA, USA).

## 3. Results and Discussion

### 3.1. Particle Size Distribution

**Figure 1:**
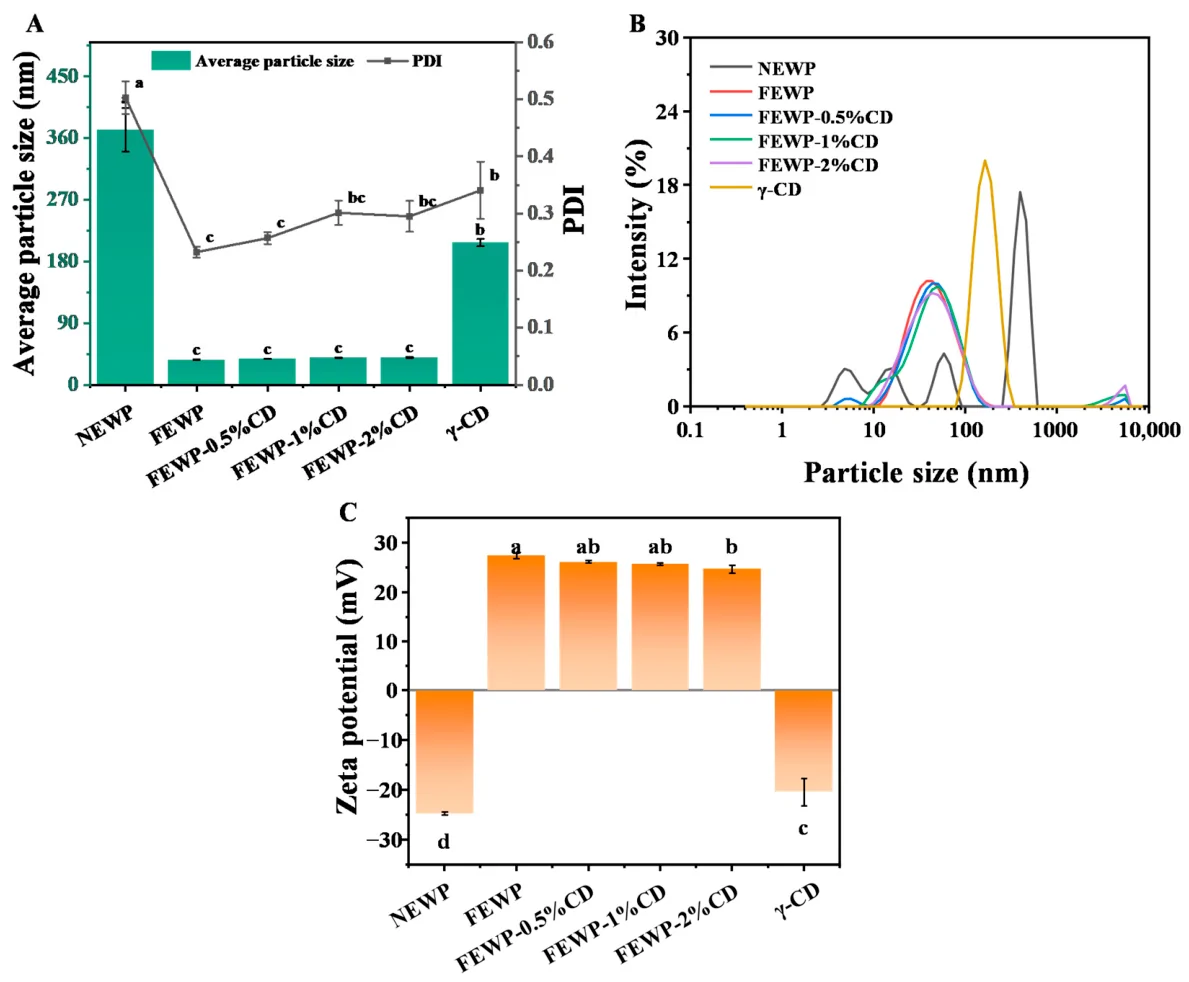
Effects of γ-cyclodextrin (γ-CD) on the colloidal characteristics of native egg white protein (NEWP) and fibrillated egg white protein (FEWP) dispersions. (**A**) Z-average particle diameter and polydispersity index (PDI); (**B**) intensity-weighted particle-size distributions; and (**C**) zeta potential. Different lowercase letters indicate significant differences among samples for the same parameter (*p* < 0.05). Sample codes indicate FEWP with the stated γ-CD concentration.

Particle size, polydispersity index (PDI), and particle size distribution collectively reflect the aggregation state and dispersion homogeneity of protein-based colloidal systems, and can therefore provide insight into the structural reorganization induced by protein fibrillation and γ-CD incorporation [19]. As shown in Figure 1A,B, NEWP exhibited a relatively large apparent hydrodynamic diameter of 371.97 ± 32.03 nm and a PDI of 0.50 ± 0.03. Its broad, multimodal particle-size distribution indicated the coexistence of several scattering populations and considerable heterogeneity within the dispersion. In contrast, acid-heat treatment substantially altered the colloidal state of NEWP. The apparent diameter of FEWP decreased to 36.56 ± 0.56 nm, accompanied by a reduction in PDI to 0.23 ± 0.01 and a pronounced narrowing of the size-distribution profile. These changes suggest that acidic heating reorganized the heterogeneous native aggregates into smaller and more uniformly dispersed species, possibly through a combination of protein unfolding, partial hydrolysis, and fibrillar assembly [15,23]. Because protein fibrils are anisotropic, however, the values obtained by dynamic light scattering should be regarded as equivalent hydrodynamic diameters rather than the actual contour lengths of individual fibrils.

The addition of γ-CD caused only a limited change in the apparent size of FEWP. Mean diameters of the FEWP-0.5%CD, FEWP-1%CD, and FEWP-2%CD systems were 37.97 ± 0.19, 39.51 ± 0.35, and 39.94 ± 0.73 nm, respectively, with no significant differences among FEWP-containing formulations (*p* > 0.05). Their distribution curves largely overlapped, while PDI increased modestly from 0.23 for FEWP to 0.26–0.30 after γ-CD incorporation. This broadening indicates a small change in the scattering population, but its molecular origin was not resolved. The absence of marked particle growth indicates that γ-CD did not produce detectable large-scale aggregation under the measurement conditions [24]. The γ-CD control showed an apparent hydrodynamic diameter of 207.73 ± 5.26 nm and a PDI of 0.34 ± 0.05. This value was substantially larger than the molecular dimensions of an individual γ-CD molecule and therefore represented the size of the scattering entities detected in the solution rather than the molecular size of γ-CD itself. Previous light-scattering and permeation studies have reported a concentration-dependent association of γ-CD in aqueous media, although the occurrence and extent of cyclodextrin aggregation remain method-dependent [25]. In the present study, the γ-CD solution was highly diluted before DLS analysis. Thus, the observed apparent diameter should not be regarded as direct evidence of extensive γ-CD self-aggregation [26]. Instead, it may reflect a small population of metastable associates or adventitious particulates, whose contribution was amplified in the intensity-weighted DLS distribution.

### 3.2. Estimated Zeta Potential

The zeta potential values were calculated from measured electrophoretic mobility using the Smoluchowski approximation and are therefore model-dependent electrokinetic estimates rather than directly measured thermodynamic potentials [16,17]. Formulation-dependent differences were observed in the estimated zeta potential (Figure 1C). NEWP exhibited a negative value of −24.73 ± 0.35 mV, whereas FEWP showed a positive value of 27.37 ± 0.50 mV, indicating that the two dispersions existed in markedly different electrokinetic states under the measurement conditions. Because the sign and magnitude of protein zeta potential depend strongly on the relationship between the dispersion pH and the protein isoelectric point [27], the observed charge reversal may reflect the combined effects of the acidic processing history, processing-induced structural reorganization, and possible residual differences in dispersion pH. As the γ-CD concentration increased, the zeta potential decreased progressively from 27.37 ± 0.50 mV for FEWP to 26.17 ± 0.21, 25.70 ± 0.20, and 24.73 ± 0.81 mV for the complexes containing 0.5%, 1%, and 2% γ-CD, respectively. The value of the FEWP-2% γ-CD system was significantly lower than that of FEWP (*p* < 0.05). Since γ-CD is a nonionic oligosaccharide, this decline was unlikely to result from direct charge neutralization. Instead, γ-CD association may have modified the hydration environment surrounding the charged groups. Comparable effects have been observed in other protein–cyclodextrin systems, in which cyclodextrin addition altered protein–protein interactions, apparent hydrophobicity, and aggregation behavior in a concentration- and protein-dependent manner [28,29]. The hydroxyl-rich outer surface of γ-CD can form extensive hydrogen-bonding interactions with water molecules, giving rise to pronounced hydration effects [30]. Such hydration may have partially compensated for the slight weakening of electrostatic repulsion by providing additional steric and hydration stabilization. This interpretation agrees with the particle-size results, which showed no marked aggregation after γ-CD incorporation. Therefore, the decrease in zeta potential should not be regarded as direct evidence of colloidal destabilization. The γ-CD solution alone exhibited an apparent zeta potential of −20.40 ± 2.75 mV. Preferential ion adsorption or trace ionic components may have contributed to the measured mobility. Overall, the particle-size and zeta-potential results indicate that γ-CD mainly modified the hydration and electrokinetic environment of FEWP without inducing extensive fibrillar aggregation.

### 3.3. UV Absorption and Second-Derivative Spectral Analysis

**Figure 2:**
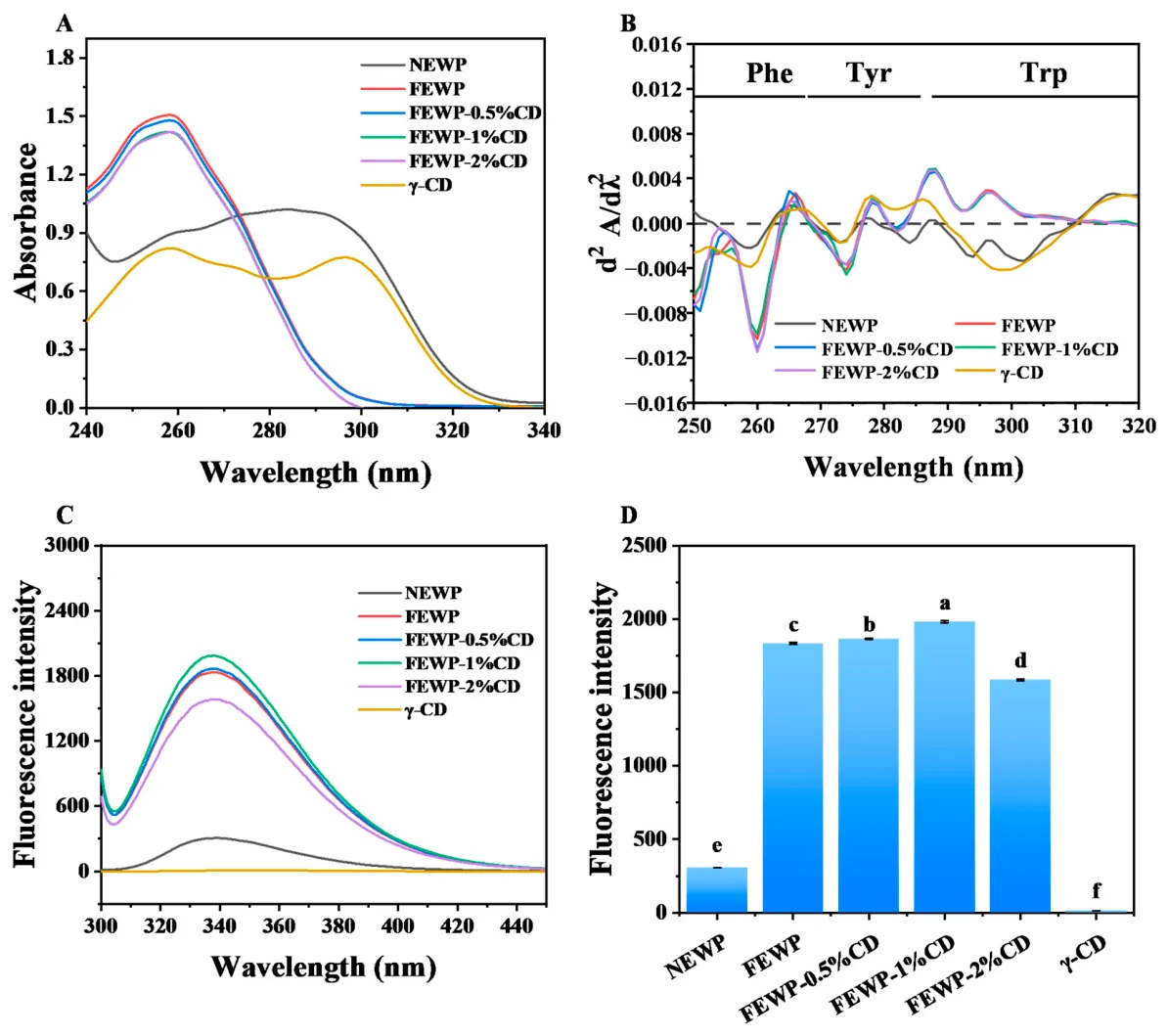
Effects of γ-cyclodextrin (γ-CD) on the aromatic-residue microenvironment of native egg white protein (NEWP) and fibrillated egg white protein (FEWP). (**A**) UV-visible absorption spectra; (**B**) second-derivative UV spectra, with the principal phenylalanine (Phe), tyrosine (Tyr), and tryptophan (Trp) regions indicated; (**C**) intrinsic fluorescence emission spectra; and (**D**) maximum intrinsic fluorescence intensity. Different lowercase letters indicate significant differences (*p* < 0.05).

As shown in Figure 2A, NEWP exhibited a broad UV absorption profile, with relatively high absorbance in the region of approximately 280–300 nm. In contrast, FEWP showed a pronounced absorption maximum at approximately 258–260 nm, followed by a rapid decline in absorbance above 270 nm. This marked alteration in spectral shape indicates that acid-heat treatment substantially changed the environment and relative spectral contributions of aromatic amino acid residues. A similar redistribution of aromatic absorption bands toward shorter wavelengths has been observed during egg-white-protein fibrillation, while studies on acid-heated ovalbumin have further associated these changes with protein unfolding, partial hydrolysis, and subsequent fibrillar self-assembly [31,32]. The spectra of the FEWP–γ-CD formulations generally resembled that of FEWP and retained similar principal absorption maxima. Only modest differences in absorbance were observed among the complexes, with no clear concentration-dependent shift in peak position.

These results suggest that γ-CD incorporation did not cause a major alteration in the overall UV spectral characteristics established during fibrillation. Comparable observations have been reported in modified protein–fibril systems, in which interactions with small molecules produced changes in absorption intensity or minor spectral shifts without completely altering the characteristic spectral profile of the fibrillar protein [33]. Because the γ-CD control itself exhibited measurable absorption in this wavelength range, the small intensity differences among the complexes were not interpreted further as direct evidence of specific inclusion or extensive conformational changes.

Second-derivative analysis, which improves the separation of overlapping aromatic absorption bands, further distinguished NEWP from the fibrillated samples (Figure 2B). NEWP displayed a derivative profile that differed markedly from those of FEWP and the FEWP-γ-CD complexes across the regions associated with phenylalanine (Phe), tyrosine (Tyr), and tryptophan (Trp). By comparison, FEWP and the three complexes showed highly similar peak and trough positions, although slight variations in amplitude were observed. Thus, acid-heat-induced fibrillation was the dominant factor affecting the aromatic-residue microenvironment, whereas γ-CD produced only limited perturbations detectable by UV spectroscopy. This interpretation is consistent with the particle-size results, which showed that the dispersed state of FEWP was largely maintained after γ-CD incorporation.

### 3.4. Intrinsic Fluorescence Spectra

Intrinsic fluorescence spectroscopy is sensitive to changes in the local environment and quenching state of aromatic residues, particularly tryptophan, and is therefore widely used to monitor protein unfolding, aggregation, and intermolecular organization [34]. As shown in Figure 2C, the γ-CD-only control produced negligible fluorescence under the measurement conditions, indicating that its direct contribution to the emission spectra of the protein-containing samples was minimal. NEWP exhibited a relatively weak emission band centered at approximately 338–340 nm, with a maximum fluorescence intensity of 306.40 ± 0.46. Following acid-heat treatment, the maximum intensity increased markedly to 1835.33 ± 7.51, corresponding to an approximately 5.99-fold enhancement relative to NEWP (*p* < 0.05). This pronounced increase indicates that acid-induced structural reorganization substantially altered the local packing and quenching environment of tryptophan residues. Under acidic heating conditions, EWPs, particularly ovalbumin, undergo conformational unfolding and partial hydrolysis, followed by the association and ordered assembly of the resulting protein species into fibrillar structures [32]. These transitions may alter the distances and interactions between tryptophan residues and neighboring quenching groups, thereby changing the balance between radiative and non-radiative relaxation pathways.

Despite the substantial increase in fluorescence intensity, the emission maximum of FEWP remained close to 338–340 nm. Acid-heat treatment therefore appeared to affect mainly the packing and quenching state of the emitting residues, without causing a major detectable change in the average polarity of their surrounding environment. The incorporation of γ-CD produced a concentration-dependent but non-monotonic fluorescence response (Figure 2D).

Relative to FEWP, the maximum intensity increased slightly but significantly to 1866.33 ± 3.06 in the FEWP-0.5%CD system and reached the highest value of 1983.67 ± 7.09 in the FEWP-1%CD system (*p* < 0.05). The increases corresponded to approximately 1.69% and 8.08%, respectively. Thus, although the change at 0.5% γ-CD was statistically significant, its magnitude was limited, whereas the 1% γ-CD treatment produced a more pronounced alteration in the local environment of the aromatic residues. Moderate γ-CD incorporation may have modified the hydration, conformational mobility, or local packing of solvent-accessible protein regions, thereby reducing some quenching interactions.

Previous studies have similarly shown that cyclodextrins can regulate protein–solvent and protein–protein interactions without necessarily inducing extensive global unfolding [35]. At 2% γ-CD, the maximum fluorescence intensity fell significantly to 1586.00 ± 4.58, representing a 13.58% decrease relative to FEWP (*p* < 0.05). This reversal may arise from changes in the accessibility and packing of aromatic residues, accompanied by shifts in local hydration and quenching conditions. Similar non-linear fluorescence responses have been observed when protein fibrils interact with low-molecular-weight modifiers, reflecting concentration-dependent rearrangement of the local aromatic environment [36,37]. Some caution is nevertheless required in interpreting the attenuation, because γ-CD showed measurable absorbance near the excitation region and an inner-filter contribution at the highest concentration cannot be ruled out. Even after this reduction, the fluorescence intensity of FEWP-2%CD remained approximately 5.18 times that of NEWP. Taken together, the fluorescence response followed a non-monotonic pattern: emission was enhanced at moderate γ-CD levels, peaking at 1%, but partially suppressed at 2%. Such changes are more consistent with localized alterations in aromatic-residue packing, hydration, and quenching than with extensive reorganization of the fibrillar architecture.

### 3.5. Emulsifying Properties

**Figure 3:**
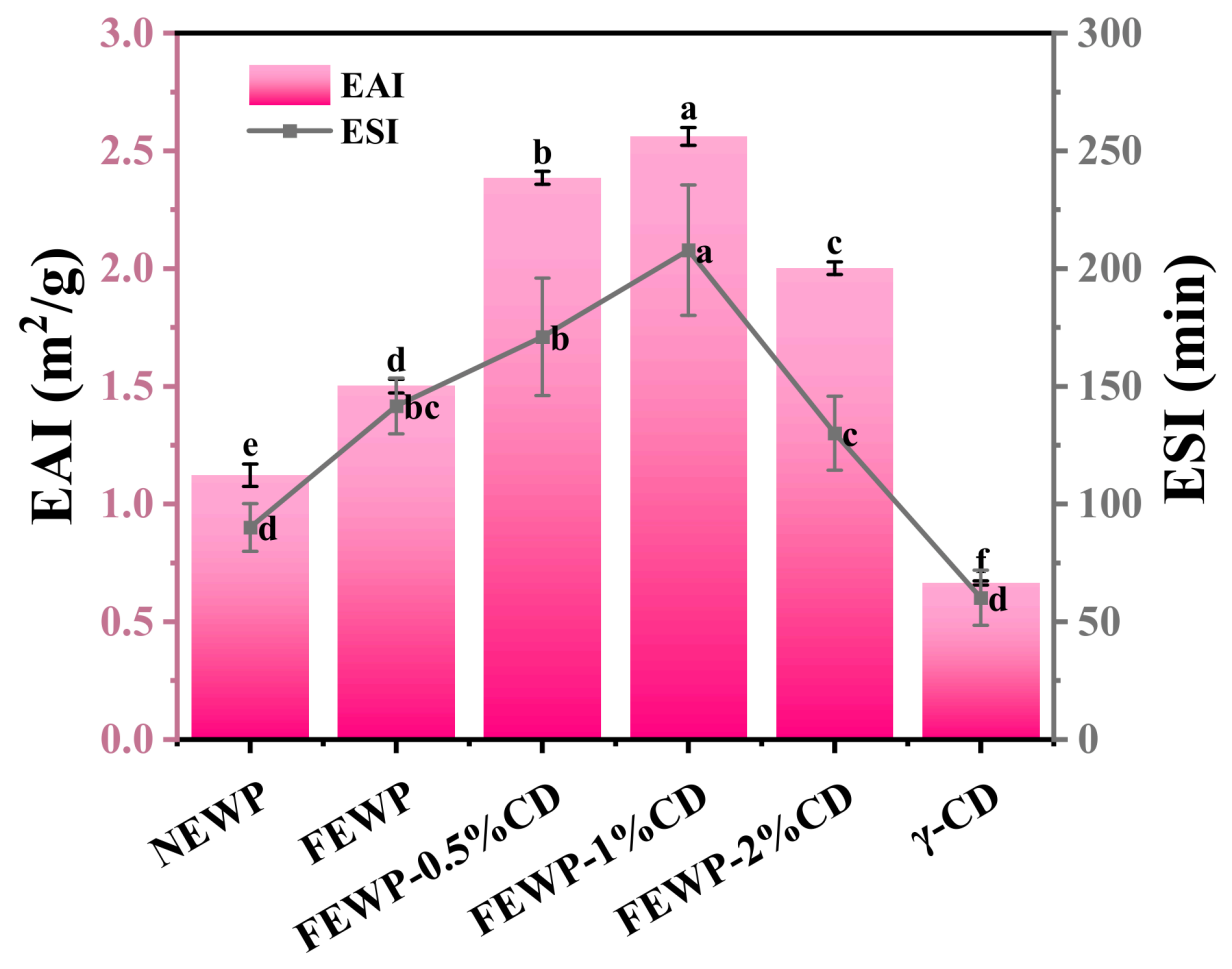
Emulsifying activity index (EAI) and emulsion stability index (ESI) of oil-in-water emulsions prepared at an aqueous-to-oil volume ratio of 70:30. Different lowercase letters indicate significant differences among samples for the same parameter (*p* < 0.05). Abbreviations: NEWP, native egg white protein; FEWP, fibrillated egg white protein; γ-CD, γ-cyclodextrin. Sample codes indicate FEWP with the stated γ-CD concentration.

The emulsifying activity index (EAI) estimates the interfacial area generated per unit mass of protein immediately after homogenization, whereas the emulsion stability index (ESI) describes the short-term retention of emulsion turbidity against droplet destabilization [37]. As depicted in Figure 3, NEWP exhibited a relatively low EAI of 1.12 ± 0.05 m^2^/g. Acid/heat treatment increased the EAI to 1.50 ± 0.03 m^2^/g (*p* < 0.05), demonstrating that fibrillation improved the ability of EWP to form a new oil–water interfacial area. Similar improvements in emulsifying functionality have been reported for food-protein fibrils and have been related to their anisotropic morphology, extended surface area, and capacity to adsorb and organize at fluid interfaces [6,8,38]. In the present study, the enhanced EAI of FEWP was also consistent with its smaller apparent hydrodynamic diameter, lower PDI, and substantially greater accessibility of hydrophobic regions than NEWP.

The incorporation of γ-CD produced a concentration-dependent but non-linear change in emulsifying activity. EAI increased significantly to 2.39 ± 0.03 and 2.56 ± 0.04 m^2^/g after the addition of 0.5% and 1% γ-CD, respectively. The FEWP-1%CD system exhibited the highest EAI, representing an increase of approximately 70.67% relative to FEWP. Moderate γ-CD addition may have weakened excessive fibril–fibril association and modified the hydration environment while retaining sufficient hydrophobic accessibility for adsorption at the oil–water interface. Direct studies on related protein–fibril systems show that fibril morphology and association state can influence adsorption and interfacial-film properties [9,39]. These reports support the plausibility of this interpretation. At 2% γ-CD, EAI decreased to 2.00 ± 0.03 m^2^/g, although it remained above that of FEWP. Saturation of preferential interaction sites or rearrangement of fibril-associated regions may have disturbed the balance between aqueous dispersibility and interfacial affinity.

A broadly similar concentration-dependent pattern was observed for ESI. Acid heat treatment increased ESI from 90.06 ± 10.14 min for NEWP to 141.65 ± 11.84 min for FEWP, indicating improved short-term emulsion stability after fibrillation. The value increased further to 171.01 ± 24.91 min with 0.5% γ-CD and reached a maximum of 207.87 ± 27.71 min in the FEWP-1%CD emulsion. In contrast, increasing the γ-CD concentration to 2% reduced ESI to 130.10 ± 15.80 min. According to the significance lettering, the FEWP-1%CD system differed significantly from all other formulations, whereas FEWP did not differ significantly from either the 0.5% or 2% γ-CD system (*p* > 0.05). The γ-CD-only emulsion showed an ESI of 60.20 ± 11.69 min, which was statistically comparable to that of NEWP, indicating that γ-CD alone provided limited short-term stabilization in the absence of protein. Overall, 1% γ-CD produced the most favorable combination of EAI and ESI. This result agrees with the particle-size and intrinsic-fluorescence results, suggesting that effective emulsification requires an appropriate balance among hydrophobic-site accessibility, fibril dispersibility, hydration, and interfacial organization. Conversely, the decline in both EAI and ESI at 2% γ-CD indicates that further addition shifted the system beyond this favorable interaction range and reduced overall emulsifying performance.

### 3.6. Microscopic Observation

**Figure 4:**
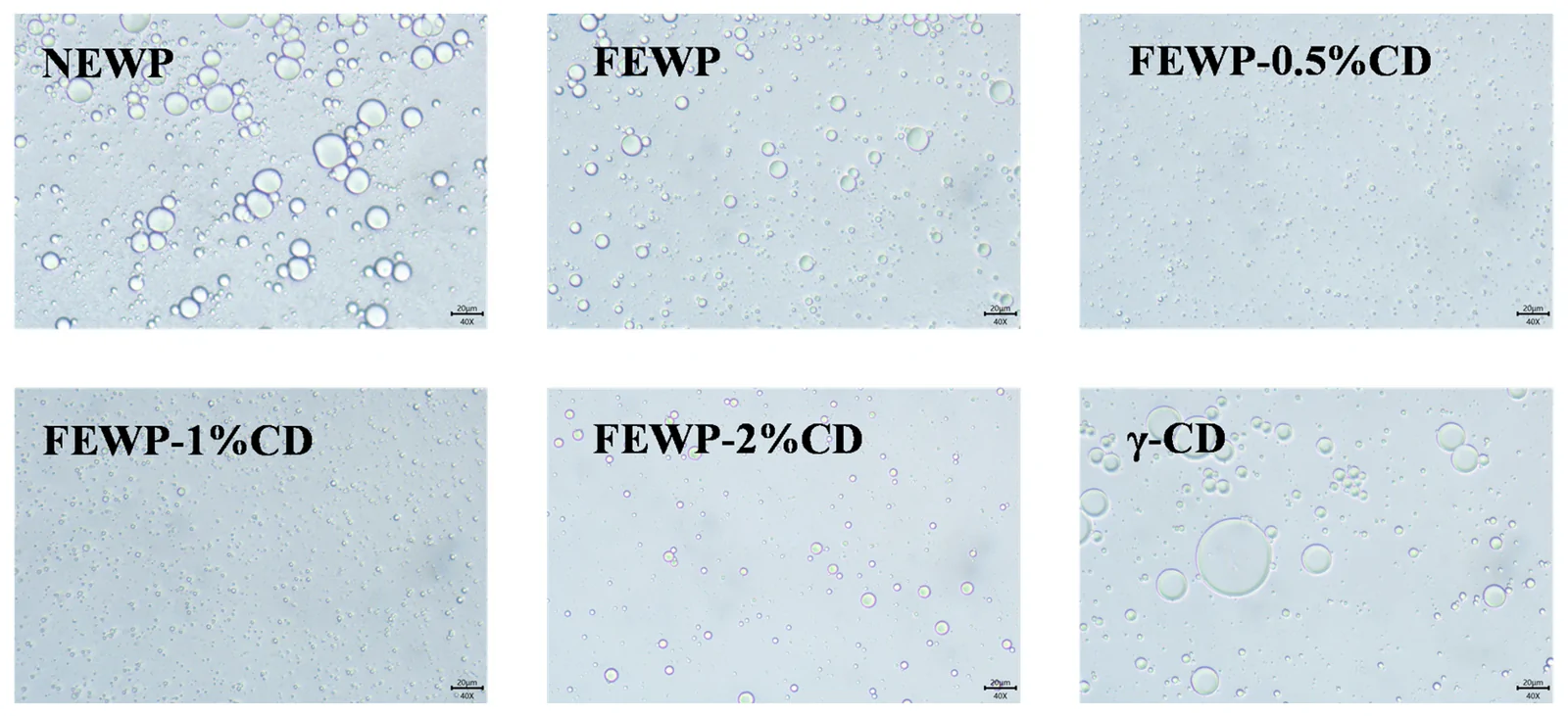
Representative optical micrographs of oil-in-water emulsions stabilized by native egg white protein (NEWP), fibrillated egg white protein (FEWP), FEWP containing 0.5%, 1%, or 2% (weight/volume) γ-cyclodextrin (γ-CD), or γ-CD alone. Images were acquired at 40× magnification under identical illumination conditions.

Qualitative examination of the optical micrographs indicated visible differences in droplet organization among the emulsions (Figure 4). The NEWP-stabilized emulsion contained numerous large droplets and showed a broad, heterogeneous distribution. Acid heat treatment noticeably reduced the prevalence of these large droplets, resulting in a finer and more uniform dispersed phase. This improvement may be related to the smaller colloidal entities and greater accessibility of hydrophobic regions generated during fibrillation, both of which could favor the interfacial organization of FEWP [6,37]. Even so, several larger droplets remained visible in the FEWP emulsion, indicating that fibrillation alone did not completely prevent localized droplet association. A further refinement of the droplet structure was observed after the incorporation of moderate amounts of γ-CD. The FEWP-0.5%CD emulsion contained a dense population of relatively small droplets, while the FEWP-1%CD system displayed the most uniform overall distribution and the fewest conspicuous large droplets. This visual pattern paralleled the superior EAI and ESI of the 1% formulation. In the FEWP-2%CD emulsion, larger droplets became more apparent and the spatial distribution was less uniform than in the 0.5% and 1% systems. The γ-CD-only sample showed the most heterogeneous droplet pattern, including several conspicuously large droplets, which indicated limited stabilization in the absence of protein.

### 3.7. Microrheological Properties

**Figure 5:**
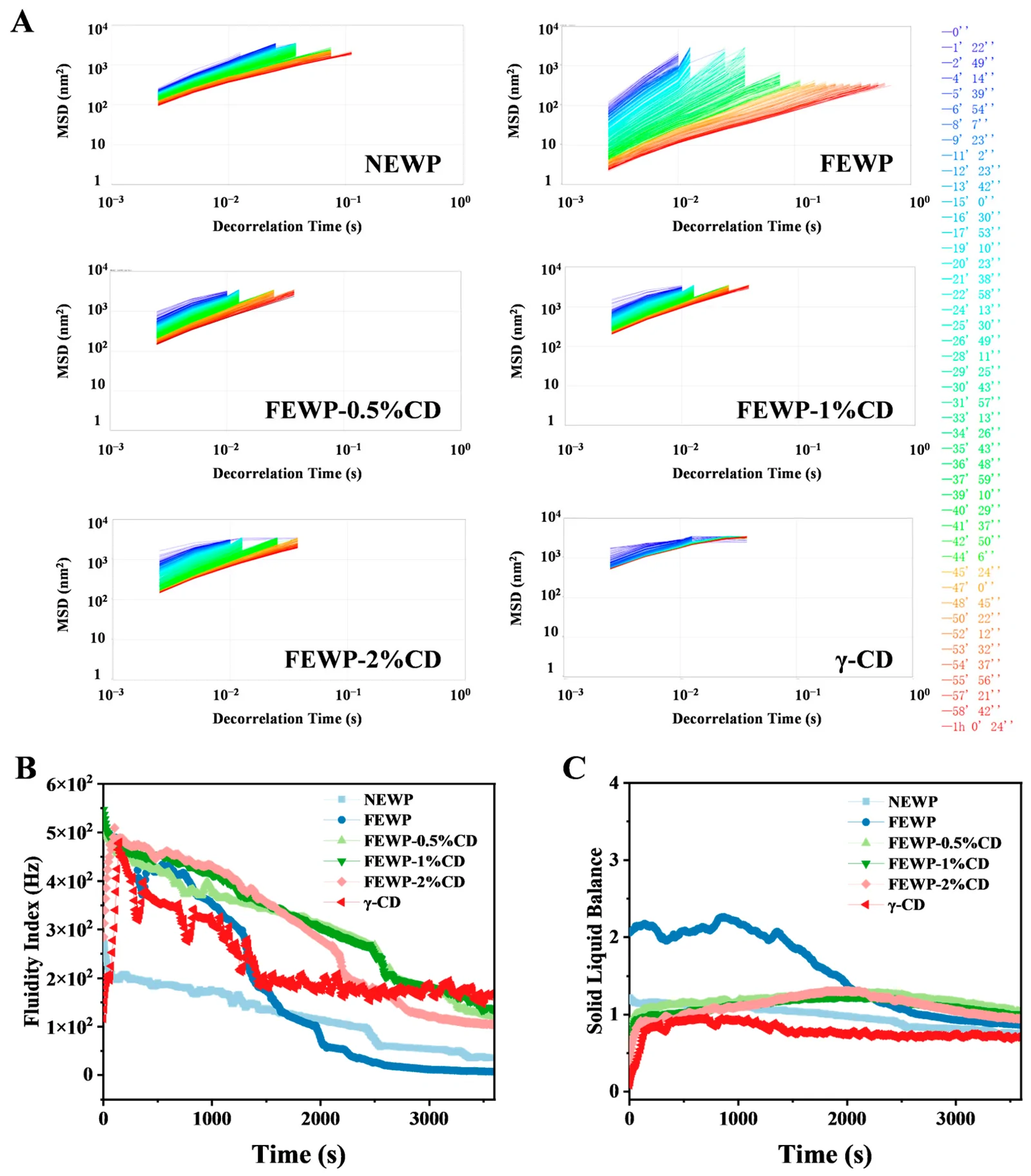
Time-dependent microrheological behavior of the emulsions during quiescent monitoring at 25 °C for 1 h. (**A**) Mean square displacement (MSD) as a function of decorrelation time; successive curves in each panel represent the evolution of the sample during the monitoring period. (**B**) Fluidity index (FI) and (**C**) solid–liquid balance (SLB) as functions of monitoring time. Abbreviations: NEWP, native egg white protein; FEWP, fibrillated egg white protein; γ-CD, γ-cyclodextrin. Sample codes indicate FEWP with the stated γ-CD concentration.

Microscopic mobility and structural evolution of the emulsions were tracked through the mean square displacement (MSD), fluidity index (FI), and solid–liquid balance (SLB) [40]. NEWP maintained relatively high MSD values throughout the 1 h monitoring period, and its FI declined only moderately, implying limited restriction of droplet motion (Figure 5A,B). A markedly different pattern emerged for FEWP. With increasing monitoring time, the MSD curves moved progressively toward lower displacement regions and FI eventually approached zero. Meanwhile, SLB initially exceeded unity and then decreased sharply (Figure 5C). This behavior reflects a highly dynamic transition from rapid, non-Brownian rearrangement to pronounced microscopic arrest, rather than the immediate establishment of a homogeneous viscoelastic network. The strong restriction of mobility in the FEWP emulsion may be associated with fibril entanglement, crowding, and fibril-mediated contacts between neighboring droplets. The elongated morphology and numerous interaction sites of protein fibrils can strengthen continuous-phase structures and impede droplet migration; once these interactions become excessive, however, bridging and localized flocculation may also occur [37,41]. Consequently, the low MSD and near-zero FI of FEWP should not be equated directly with superior emulsion stability. Instead, they indicate progressive immobilization within a structurally evolving system.

Compared with FEWP, the γ-CD-containing emulsions showed less widely separated successive MSD curves, higher FI values, and more gradual SLB changes during quiescent monitoring. These features point to a more stable mobility pattern under quiescent conditions. Similar moderation of droplet motion has been reported in protein-biopolymer emulsions, where enhanced hydration and steric effects altered the development of microscopic structure [27,41]. The FEWP-1%CD formulation occupied an intermediate mobility state between the highly mobile NEWP system and the strongly arrested FEWP emulsion, and this pattern was considered together with its high EAI and ESI and the qualitative microscopy observations.

### 3.8. Rheological Measurements

**Figure 6:**
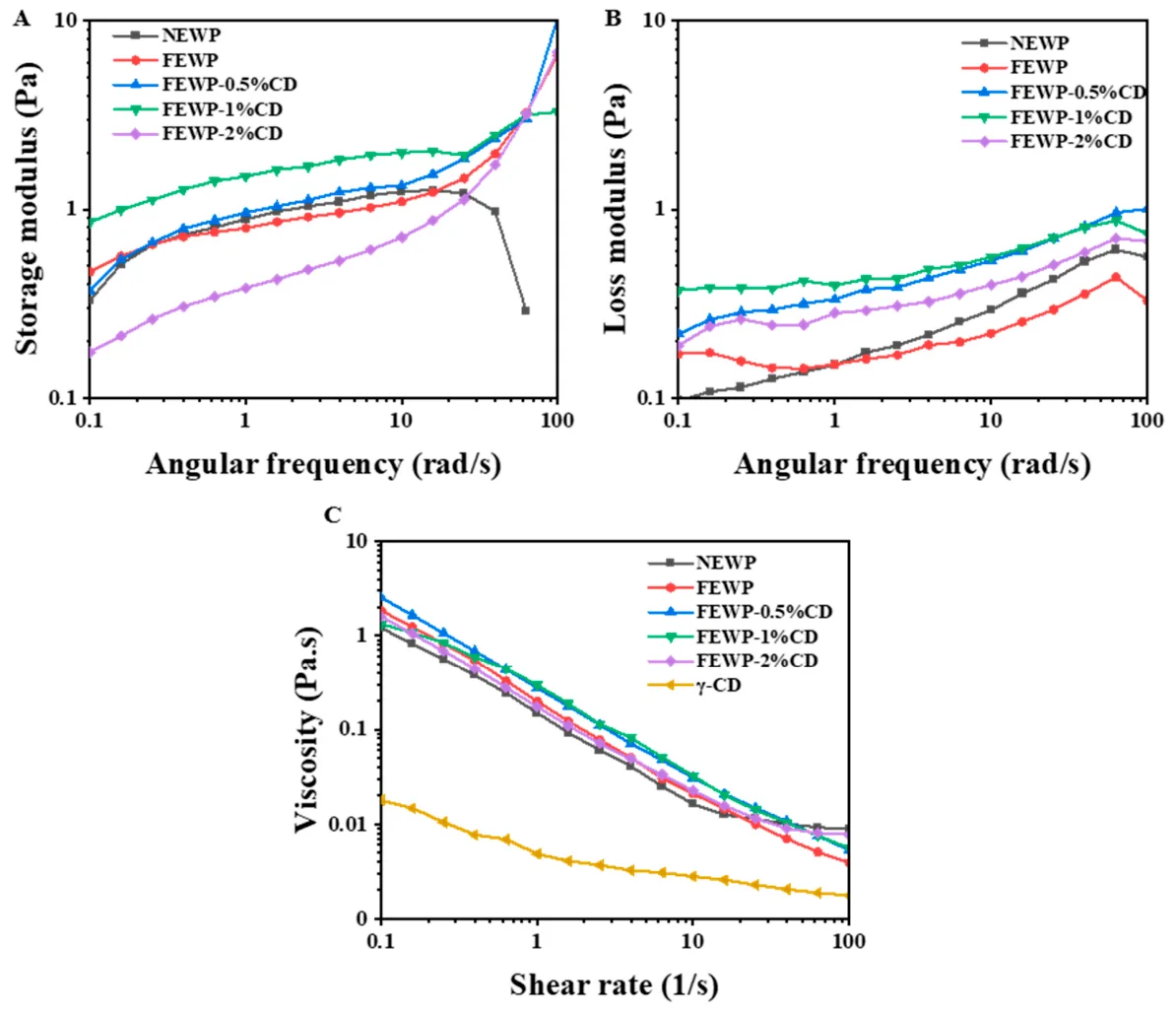
Dynamic oscillatory and steady-shear rheological properties of the emulsions at 25 °C. (**A**) Storage modulus (G’) and (**B**) loss modulus (G″) as functions of angular frequency at 1% strain, and (**C**) apparent viscosity as a function of shear rate. Abbreviations: NEWP, native egg white protein; FEWP, fibrillated egg white protein; γ-CD, γ-cyclodextrin. Sample codes indicate FEWP with the stated γ-CD concentration.

Across the reliable frequency range, G’ remained above G″ in all protein-stabilized emulsions (Figure 6A,B), demonstrating an elastic-dominated viscoelastic response [3]. Recent studies on fibril-containing emulsions and emulsion gels have similarly shown that fibril morphology, interfacial adsorption, and interactions among dispersed droplets can collectively determine the development of long-timescale structural connectivity [10,42]. In the present system, FEWP-1%CD exhibited comparatively high G’ and G″ values at low and intermediate frequencies, followed by FEWP-0.5%CD. The elevated moduli might suggest that moderate γ-CD incorporation facilitated effective coupling among the fibrillar species, adsorbed interfacial layers, and oil droplets, although frequency-sweep data alone cannot establish the presence of a permanent continuous network. The lower low-frequency G’ of FEWP-2%CD, by comparison, indicates that the structural connections resisting slow deformation were less effectively maintained. The dependence of emulsion viscoelasticity on fibril morphology and interfacial organization has also been suggested in recent studies of ovalbumin- and soy-protein-derived fibrils [6,9].

Steady-shear measurements offered a complementary view of the structural response under flow (Figure 6C). Apparent viscosity decreased continuously with increasing shear rate in all formulations, confirming pronounced shear-thinning behavior. Comparable flow characteristics have recently been reported for protein–fibril-containing emulsion gels and amyloid-fibril-stabilized high-internal-phase emulsions, where fibrillar entanglement and droplet association increased resistance to flow under weak shear but were progressively disrupted or oriented as shear intensified [42,43]. The FEWP-0.5%CD and FEWP-1%CD emulsions displayed the highest viscosities in the low-shear region, whereas FEWP remained more viscous than NEWP. These differences show that fibrillation strengthened bulk-phase structuring and that moderate γ-CD incorporation further reinforced the weak associations prevailing under near-quiescent conditions. FEWP-2%CD showed a less pronounced viscosity increase, possibly because changes in fibril hydration and spatial organization reduced effective fibril-droplet coupling. At higher shear rates, the viscosity curves gradually converged as these structures were disrupted by the imposed flow. Thus, FEWP-1%CD retained appreciable resistance to weak deformation while remaining readily shearable at elevated shear rates.

### 3.9. Creep-Recovery and Thixotropic Behavior

**Figure 7:**
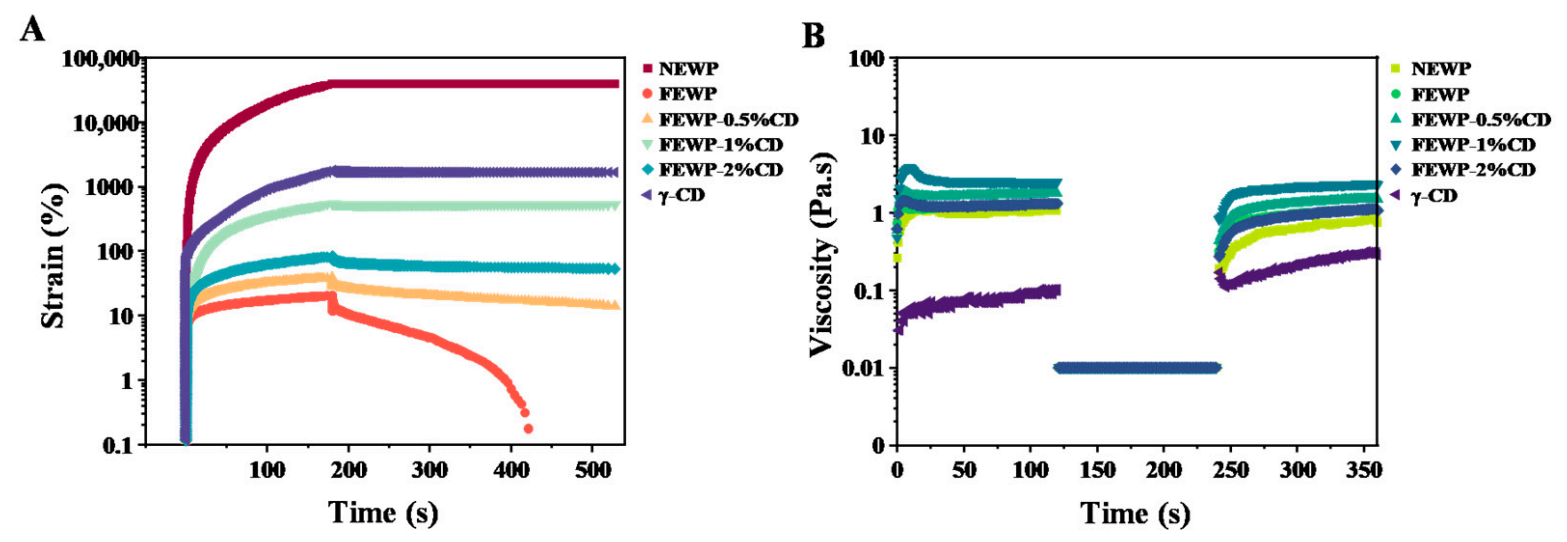
Creep-recovery and thixotropic behavior of the emulsions at 25 °C. (**A**) Strain response during 180 s of creep followed by 360 s of recovery after removal of the applied stress, and (**B**) apparent viscosity during a three-interval thixotropy test consisting of 0.1 s^−1^ for 120 s, 100 s^−1^ for 120 s, and 0.1 s^−1^ for 120 s. Abbreviations: NEWP, native egg white protein; FEWP, fibrillated egg white protein; γ-CD, γ-cyclodextrin. Sample codes indicate FEWP with the stated γ-CD concentration.

Creep-recovery testing differentiated the emulsions according to their response to sustained loading (Figure 7A). FEWP accumulated the least strain during the creep stage and underwent substantial recovery after unloading, revealing a rigid and strongly arrested structure with an appreciable recoverable contribution [44]. Its mechanical response echoed the microrheological signature characterized by lower MSD and a progressive loss of fluidity. Nevertheless, the limited deformation of FEWP should not be regarded as direct evidence of a uniformly organized or inherently more stable emulsion. Entanglement of elongated fibrils, fibril-mediated bridging between droplets, and localized crowding can all suppress deformation and generate an apparently elastic bulk response [6,43]. The FEWP-1%CD emulsion was more compliant under the same loading conditions while retaining clear recovery after stress removal. Such deformability does not conflict with its high EAI and ESI or its relatively homogeneous droplet distribution. Creep behavior primarily reflects the rigidity and recoverability of the bulk structure, whereas EAI, ESI, and microscopic morphology describe interfacial formation and short-term droplet stabilization. The results therefore distinguish the highly arrested FEWP emulsion from the more deformable but effectively stabilized structure formed in the presence of 1% γ-CD.

A sharp loss of apparent viscosity occurred in every formulation during the high-shear interval of the three-interval thixotropy test (Figure 7B). After restoration of the initial low shear rate, all formulations showed partial viscosity rebuilding. FEWP-1%CD had the highest absolute viscosity in both low-shear intervals, closely followed by FEWP-0.5%CD. These curves show that the 1% formulation returned to a comparatively high post-shear viscosity, although a normalized recovery ratio would be required to establish whether it also achieved the greatest proportional recovery [45]. FEWP-2%CD showed a lower viscosity during the final low-shear interval. These measurements characterize macroscopic viscosity rebuilding but cannot directly resolve hydration changes or specific load-bearing contacts. The distinctive measured features of FEWP-1%CD were therefore moderate creep compliance and a comparatively high absolute post-shear viscosity.

## 4. Possible Mechanism of γ-CD-Mediated Regulation of FEWP Emulsions

**Figure 8:**
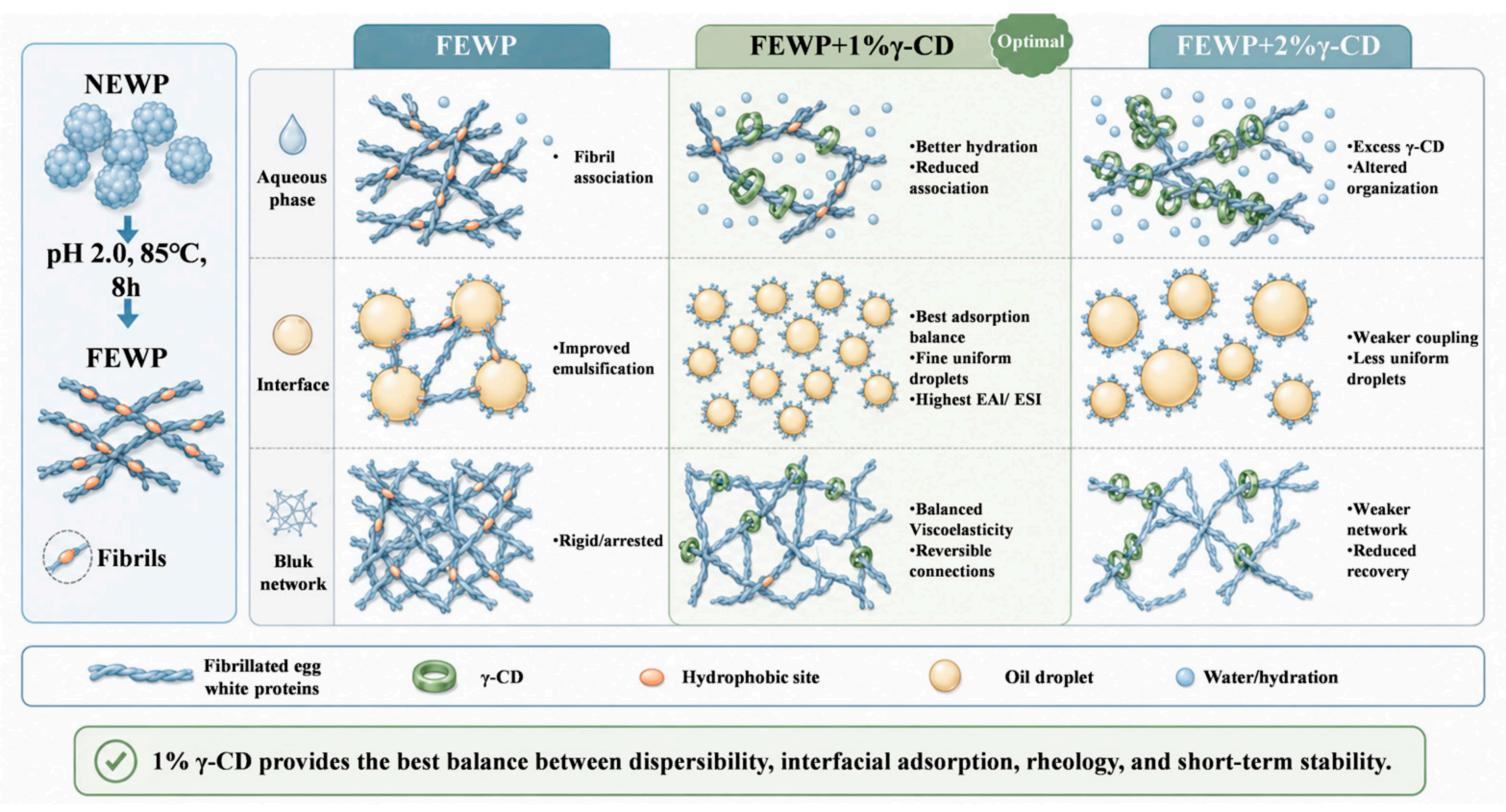
Proposed mechanism of γ-cyclodextrin (γ-CD)-mediated regulation of fibrillated egg white protein (FEWP) in oil-in-water emulsions. Additional abbreviations: NEWP, native egg white protein; EAI, emulsifying activity index; ESI, emulsion stability index.

Compared with NEWP, redispersed FEWP showed a smaller equivalent hydrodynamic diameter, higher EAI, and stronger microscopic and bulk immobilization. γ-CD produced only small particle-size changes but altered the intrinsic-fluorescence response in a non-monotonic manner. At 1% γ-CD, the measured EAI and ESI were highest; the observed microscopic fields contained fewer conspicuous large droplets; and microrheological and bulk rheological measurements indicated less progressive arrest than FEWP alone while retaining appreciable low-shear structure. These associations are compatible with concentration-dependent changes in FEWP organization and local solvent interactions. A schematic summary of this proposed interpretation is presented in Figure 8.

At 2% γ-CD, EAI and ESI were lower than at 1%, more conspicuous large droplets were observed, and the bulk rheological response indicated weaker structural connectivity. The response at 2% γ-CD indicates that this regulation occurred within a finite concentration range. Further addition may have altered the spatial distribution and persistence of hydrophobic contacts and hydration layers after preferential interaction regions approached saturation. These changes may have reduced cooperative coupling among fibrils, droplets, and interfacial films, thereby weakening both interfacial organization and bulk structural connectivity. Therefore, the concentration-dependent mechanism can be regarded as a gradual transition: 0.5% γ-CD was consistent with partial moderation of fibrillar association, whereas 1% showed the most favorable overall balance among the measured properties.

## 5. Conclusions and Future Perspectives

This study identified a concentration-dependent association between γ-CD addition and the short-term emulsifying and rheological properties of FEWP emulsions. Relative to NEWP, redispersed FEWP showed a smaller equivalent hydrodynamic diameter, higher EAI, and stronger bulk immobilization. γ-CD caused no marked particle growth and produced a non-monotonic intrinsic-fluorescence response. Among the levels tested, 1% γ-CD gave the highest EAI and 30 min ESI, fewer conspicuous large droplets in the observed microscopic fields, and a favorable combination of microrheological and bulk rheological behavior. The 2% formulation showed lower emulsifying performance and weaker structural connectivity than the 1% formulation. Thus, 1% γ-CD was the most favorable of the tested concentrations for freshly prepared emulsions. Future research should build on this concentration-dependent framework by examining whether the favorable effects of γ-CD persist during storage and in application-oriented food systems. Integrating protein organization, interfacial behavior, droplet evolution, and rheological stability will provide a more comprehensive understanding of γ-CD regulation across multiple structural levels. This progression will support formulation refinement and the practical application of FEWP-based emulsions in food products.

## Data Availability

The original contributions presented in this study are included in the article. Further inquiries can be directed to the corresponding authors.

## Abbreviations

DLS	Dynamic light scattering
EAI	Emulsifying activity index
ESI	Emulsion stability index
EWP	Egg white protein
FEWP	Fibrillated egg white protein
FI	Fluidity index
G′	Storage modulus
G″	Loss modulus
MSD	Mean square displacement
NEWP	Native egg white protein
PDI	Polydispersity index
SLB	Solid–liquid balance
UV-Vis	Ultraviolet-visible spectroscopy
γ-CD	γ-Cyclodextrin

## References

[1] Razi S.M., Fahim H., Amirabadi S., Rashidinejad A. (2023). An overview of the functional properties of egg white proteins and their application in the food industry. Food Hydrocoll..

[2] Zang J., Qing M., Chi Y., Chi Y. (2025). Egg white protein under thermal stress: Thermal aggregation orientation and gel properties decline. Int. J. Biol. Macromol..

[3] Li X., Wu M., Xia M., Salama M., Sun H., Ding L., Huang X., Shu D., Cai Z. (2024). A promising food-grade protector for Retinyl acetate emulsions with fibrillated egg white. Food Chem..

[4] Alavi F., Emam-Djomeh Z., Mohammadian M., Salami M., Moosavi-Movahedi A.A. (2020). Physico-chemical and foaming properties of nanofibrillated egg white protein and its functionality in meringue batter. Food Hydrocoll..

[5] Zhang Y., Lv X., Abker A.M., Oh D.-H., Mohamed Kassem J., Salama M., Fu X. (2024). Research progress of protein fibrils: A review of formation mechanism, characterization and applications in the food field. Food Hydrocoll..

[6] Huyst A.M.R., Luyckx T., Monge-Morera M., Van Camp J., Delcour J.A., Van der Meeren P. (2025). Interfacial and oil-in-water emulsifying properties of ovalbumin enriched in amyloid-like fibrils and peptides. Food Hydrocoll..

[7] Jiang F., Pan Y., Peng D., Huang W., Shen W., Jin W., Huang Q. (2022). Tunable self-assemblies of whey protein isolate fibrils for pickering emulsions structure regulation. Food Hydrocoll..

[8] Xu Y., Ma C.-M., Yang Y., Bian X., Liu X.-F., Wang Y., Zhang N. (2023). Food-derived protein amyloid-like fibrils: Fibrillation mechanism, structure, and recent advances for the stabilization of emulsions. Food Hydrocoll..

[9] Zhao J., Chang B., Hu Y., Wang X., Cao Z., Zhang Y., Xu Z., Sui X. (2025). Adsorption mechanism of soy protein amyloid fibrils with different morphological structures at the interface of oil-in-water emulsion. Food Hydrocoll..

[10] Cheng C., Xu Q., Li Y., Haubruge E., Liao N., Zhu G., Liu K. (2026). Impact of pH shifting on structural and emulsifying characteristics of whey protein isolate amyloid fibrils. LWT.

[11] Zhang Y.-h., Lin Y., Lin Y., Luo Z., Li Z., Wu Y.-L. (2025). Redefining cyclodextrin-based systems: A multiscale framework of structure, function and application interconnection. Chem. Eng. J..

[12] Xiao Z., Xu Z., Zhou L., Kang Y., Niu Y., Zhao D. (2025). Application of cyclodextrin-based microcapsules in food flavors and fragrances. Carbohydr. Polym..

[13] Zhang J., Zhu R., Zou B., Zheng X., Zhang R., Na X., Xu X., Du M., Wu C. (2025). β-Cyclodextrin/resistant dextrin induced disparate gelling behaviors of high-protein liquid systems. Carbohydr. Polym..

[14] Li H., Gao F., Wang X., Han Y., Lei W., Gao Z. (2025). Ultrasonic cavitation-driven construction of hydroxypropyl-β-cyclodextrin and whey protein co-assembled nanoparticles: Loading, sustained-release delivery and interaction mechanisms for EGCG. Food Chem..

[15] Dong W., Zhang X., Ding L., Liu C., Ai M., Jin Y., Isobe K., Handa A., Cai Z. (2023). Enhancement of emulsification properties by modulation of egg white protein fibril structure with different heating times. Food Hydrocoll..

[16] Gopmandal P.P., Duval J.F.L. (2022). Electrostatics and electrophoresis of engineered nanoparticles and particulate environmental contaminants: Beyond zeta potential-based formulation. Curr. Opin. Colloid Interface Sci..

[17] Bhattacharjee S. (2016). DLS and zeta potential—What they are and what they are not?. J. Control. Release.

[18] Jin H., Jin Y., Pan J., Sun Y., Sheng L. (2022). Multidimensional evaluation of structural properties of ovalbumin at the air-water interface: Spectroscopy and molecular dynamics simulations. Food Hydrocoll..

[19] Li X., Hu W., Li L., Salama M., Chen J., Deng L., Sheng L., Cai Z. (2026). Regulation of solubility and thermal stability for egg white proteins via alkaline heat treatment: A new insight into protein conformation and interactions at the nanoscale. Food Chem..

[20] Salama M., Gouda M., Abou-Elsoud M., Shu D., Li X., Sheng L., Cai Z. (2025). Insight into dairy casein implications that enhance egg white proteins’ thermodynamic and microrheological functions. Food Chem..

[21] Li X., Salama M., Chen J., Mi S., Shu D., Sheng L., Cai Z. (2026). Combined γ-cyclodextrin and trehalose regulate thermal aggregation and volatile profiles in alkaline heat-pretreated egg white protein systems. Food Hydrocoll..

[22] Liang W., Xie J., Zhao Q., Jiang C., Zhan X., Chen M., Zhang L., Li C., Rong J., Hu Y. (2026). One stone, two birds: Preparation of nutritionally fortified and easy-to-swallow 3D printed pea protein gel based on high internal phase emulsions. Food Hydrocoll..

[23] Li J., Wang Z., Yang B., Peng X., Liu S., Sun N., Su R., Tong X., Wang H., Jiang L. (2026). Time and pH regulation of the morphology and structure evolution of soybean protein fibrils: Focusing on foaming mechanism and application. Food Hydrocoll..

[24] Aachmann F.L., Larsen K.L., Wimmer R. (2012). Interactions of cyclodextrins with aromatic amino acids: A basis for protein interactions. J. Incl. Phenom. Macrocycl. Chem..

[25] Valente A.J.M., Carvalho R.A., Söderman O. (2015). Do Cyclodextrins Aggregate in Water? Insights from NMR Experiments. Langmuir.

[26] Ryzhakov A., Do Thi T., Stappaerts J., Bertoletti L., Kimpe K., Sá Couto A.R., Saokham P., Van den Mooter G., Augustijns P., Somsen G.W. (2016). Self-Assembly of Cyclodextrins and Their Complexes in Aqueous Solutions. J. Pharm. Sci..

[27] Yang Z., Li Z., Xu Z., Kong Z., Qiao X., Zhang L., Dai L., Wang Y., Sun Q., McClements D.J. (2023). Properties of Heat-Assisted pH Shifting and Compounded Chitosan from Insoluble Rice Peptide Precipitate and Its Application in the Curcumin-Loaded Pickering Emulsions. Foods.

[28] Stolzke T., Krieg F., Peng T., Zhang H., Häusler O., Brandenbusch C. (2022). Hydroxylpropyl-β-cyclodextrin as Potential Excipient to Prevent Stress-Induced Aggregation in Liquid Protein Formulations. Molecules.

[29] Johann F., Wöll S., Gieseler H. (2024). Evaluating the Potential of Cyclodextrins in Reducing Aggregation of Antibody–Drug Conjugates with Different Payloads. J. Pharm. Sci..

[30] Pereva S., Dobrev S., Sarafska T., Nikolova V., Angelova S., Spassov T., Dudev T. (2024). Deciphering the mechanism of γ-cyclodextrin’s hydrophobic cavity hydration: An integrated experimental and theoretical study. Beilstein J. Org. Chem..

[31] Bharmoria P., Mondal D., Pereira M.M., Neves M.C., Almeida M.R., Gomes M.C., Mano J.F., Bdikin I., Ferreira R.A.S., Coutinho J.A.P. (2020). Instantaneous fibrillation of egg white proteome with ionic liquid and macromolecular crowding. Commun. Mater..

[32] Chang C., Li X., Li J., Su Y., Gu L., Xiong W., Yang Y. (2023). Fabrication mechanism and functional properties of ovalbumin fibrils prepared by acidic heat treatment. J. Sci. Food Agric..

[33] Tong X., Cao J., Tian T., Lyu B., Miao L., Lian Z., Cui W., Liu S., Wang H., Jiang L. (2022). Changes in structure, rheological property and antioxidant activity of soy protein isolate fibrils by ultrasound pretreatment and EGCG. Food Hydrocoll..

[34] Salama M., Gouda M., Abou-Elsoud M., Li X., Zhang X., Sheng L., Cai Z. (2025). Physicochemical integration of egg white proteins and milk casein based on phase separation as a stable and optimized colloidal complex. Food Hydrocoll..

[35] Rospiccio M., Arsiccio A., Winter G., Pisano R. (2021). The Role of Cyclodextrins against Interface-Induced Denaturation in Pharmaceutical Formulations: A Molecular Dynamics Approach. Mol. Pharm..

[36] Xu Z., Shan G., Hao N., Li L., Lan T., Dong Y., Wen J., Tian R., Zhang Y., Jiang L. (2022). Structure remodeling of soy protein-derived amyloid fibrils mediated by epigallocatechin-3-gallate. Biomaterials.

[37] Yue J., Yao X., Gou Q., Li D., Liu N., Yang D., Gao Z., Midgley A., Katsuyoshi N., Zhao M. (2022). Recent advances of interfacial and rheological property based techno-functionality of food protein amyloid fibrils. Food Hydrocoll..

[38] Qi X., Lv X., Pan W., Shen M., Chen Y., Yu Q., Xie J. (2024). Antioxidant amyloid fibril derived from rice protein hydrolysate as stabilizer towards preparing high-stable emulsion. Food Chem..

[39] Hou J., Xu H.-N. (2025). Microfluidic droplet analysis on heterogeneous interfacial films composed of cyclodextrin and sodium casein where stiff meets soft. Food Hydrocoll..

[40] Salama M., Gouda M., Abou-Elsoud M., Li X., Ali S., Sheng L., Cai Z. (2026). Ultrasound-assisted xylitol conjugation improves heat resistance of egg white-casein complexes for food colloidal applications. Food Hydrocoll..

[41] Wei Z., Dong Y., Si J. (2024). Ovotransferrin Fibril—Gum Arabic Complexes as Stabilizers for Oleogel-in-Water Pickering Emulsions: Formation Mechanism, Physicochemical Properties, and Curcumin Delivery. Foods.

[42] Lin Y., Roos Y.H., Miao S. (2025). The effect of whey/soy protein fibril systems on the properties of fish gelatin stabilized emulsion gels. Food Hydrocoll..

[43] Han T., Guo R., An Y., Huang Y., Liu L., Wang Z., Zhu Y., Zhu X. (2026). High internal phase emulsions stabilised by amyloid fibrils from 7S/11S soybean protein at varying ratios: Study on interfacial behaviour, rheological properties and curcumin delivery potential. Food Chem..

[44] Zheng L.y., Li D., Wang L.j. (2025). Rheology and printability of biopolymeric oil-in-water high internal phase Pickering emulsions: A review. Compr. Rev. Food Sci. Food Saf..

[45] An Y., Zhu Y., Xia X., Huang Y., Lv M., Zhu X. (2026). Structural properties, rheological characteristics, and drug delivery efficiency of resveratrol-loaded soybean protein isolate and hemp protein isolate emulsion gels: Synergistic effects of carboxymethyl cellulose incorporation and ultrasound treatment. Food Chem. X.

